# Signs of Maturity in New-born Children

**Published:** 1843-01

**Authors:** 


*Signs of Maturity in new-born Children.*—According to Chaussier, if a mature child be measured immediately after birth, the middle of its length will be exactly at the navel, or a very little below. It is, however, doubtful whether this happens in all cases; and Mende has impugned its general accuracy. The following are some results noticed by Mr. Taylor, Lecturer on Medical Jurisprudence at Guy's Hospital, and Dr. Geoghegan, Professor of Med. Jurisp. in the Royal College of Surgeons in Ireland.

| Case. | Whole length. | Attachment of the Umbilical Cord. |
|---|---|---|
| 1. | 18½ | a quarter of an inch below the centre. |
| 2. | 20 | half an inch " " " |
| 3. | 17½ | half an inch nearly " " |
| 4. | 16½ | half an inch " " |
| 5. | 19 | half an inch " " |
| 6. | 17 | a little below " " |
| 7. | 18 | exactly at the centre. |
| 8. | 17 | exactly at the centre. |
| 9. | 20¾ | a little below. |
| 10. | 19½ | a little below. |
| 11. | 18¾ | exactly at the centre. |

*Guy's Hospital Reports*, April, 1842.

